# West Nile Virus Vaccination Protects against Usutu Virus Disease in Mice

**DOI:** 10.3390/v13122352

**Published:** 2021-11-23

**Authors:** Rebecca Salgado, Seth A. Hawks, Francesca Frere, Ana Vázquez, Claire Y.-H. Huang, Nisha K. Duggal

**Affiliations:** 1 Department of Biomedical Sciences and Pathobiology, Virginia-Maryland College of Veterinary Medicine, Virginia Polytechnic Institute and State University, Blacksburg, VA 24061, USA; rsalgado97@vt.edu (R.S.); sah1026@vt.edu (S.A.H.); ffrere@vt.edu (F.F.); 2 National Centre for Microbiology, Instituto de Salud Carlos III (ISCIII), CIBERESP, CIBER Epidemiology and Public Health, 28220 Madrid, Spain; a.vazquez@isciii.es; 3 Arboviral Diseases Branch, Centers for Disease Control and Prevention, Division of Vector-Borne Diseases, Fort Collins, CO 80521, USA; yxh0@cdc.gov

**Keywords:** flavivirus, vaccine, West Nile virus, Usutu virus, neutralizing response

## Abstract

West Nile virus (WNV) and Usutu virus (USUV) are mosquito-borne flaviviruses that can cause neuroinvasive disease in humans. WNV and USUV circulate in both Africa and Europe and are closely related. Due to antigenic similarity, WNV-specific antibodies and USUV-specific antibodies have the potential to bind heterologous viruses; however, it is unclear whether this interaction may offer protection against infection. To investigate how prior WNV exposure would influence USUV infection, we used an attenuated WNV vaccine that contains the surface proteins of WNV in the backbone of a dengue virus 2 vaccine strain and protects against WNV disease. We hypothesized that vaccination with this attenuated WNV vaccine would protect against USUV infection. Neutralizing responses against WNV and USUV were measured in vitro using sera following vaccination. Sera from vaccinated CD-1 and *Ifnar1^−/−^* mice cross-neutralized with WNV and USUV. All mice were then subsequently challenged with an African or European USUV strain. In CD-1 mice, there was no difference in USUV titers between vaccinated and mock-vaccinated mice. However, in the *Ifnar1^−/−^* model, vaccinated mice had significantly higher survival rates and significantly lower USUV viremia compared to mock-vaccinated mice. Our results indicate that exposure to an attenuated form of WNV protects against severe USUV disease in mice and elicits a neutralizing response to both WNV and USUV. Future studies will investigate the immune mechanisms responsible for the protection against USUV infection induced by WNV vaccination, providing critical insight that will be essential for USUV and WNV vaccine development.

## 1. Introduction

West Nile virus (WNV) and Usutu virus (USUV) are emerging zoonotic arboviruses in the Japanese encephalitis virus (JEV) serocomplex of the *Flaviviridae* family. Clinical manifestations of WNV and USUV in humans include febrile illness and neuroinvasive disease, which in severe cases can be fatal. WNV and USUV are maintained in a transmission cycle between *Culex* spp. mosquito vectors and avian hosts [1]. WNV was first isolated in 1937 from a febrile patient in Uganda [2] and circulated throughout Africa [3,4,5], Asia [6,7,8], Australia [9,10], the Americas [11,12,13], and Europe [14,15,16]. USUV was originally isolated in South Africa in 1959 [17] and circulated throughout Africa since that time, eventually spreading to Europe [18]. Shortly after the introduction of WNV into North America in 1999, the first major epizootic event of USUV occurred in Europe in 2001, where approximately 50,000 Eurasian blackbirds (*Turdus merula*) died [19,20].

WNV and USUV have overlapping geographic ranges and transmission cycles, thus having the potential to infect the same host. In humans, both WNV- and USUV-specific antibodies were found in healthy blood donors in Italy [21,22]. Evidence of sequential WNV and USUV infections in humans was also observed; during a WNV outbreak in 2018, individuals with prior USUV exposure had an atypical antibody response to WNV, characterized by an absent or blunt WNV IgM response [23]. Due to antigenic similarity between WNV and USUV, cross-neutralizing antibody responses were studied. In Austria, sera from confirmed WNV-infected individuals neutralized both WNV and USUV in vitro at different titers [24]. Additionally, cross-protection between WNV and USUV was modeled experimentally; one study observed that mice infected with USUV were protected from lethal WNV challenge [25]. However, whether exposure to WNV offers protection from USUV infection is unclear.

A recombinant live-attenuated vaccine (LAV) virus, D2/WN-V3 (also referred as D2/WN for abbreviation) that protects against lethal challenge of WNV in mice was previously developed, consisting of the premembrane (prM) and envelope (E) structural genes of WNV in an attenuated dengue virus (D2) backbone [26]. The D2/WN LAV retained all the original attenuation markers of the D2 backbone [27,28,29] and protected against lethal WNV challenge in vivo, including diminished neurovirulence in newborn mice and development of neutralizing antibodies against WNV in adult mice after primary immunization [26]. D2/WN was also evaluated for safety in mice, with no disease observed in newborn mice or AG129 mice [26,30].

The goal of this study was to determine if prior exposure to the WNV LAV would protect against subsequent USUV infection. For our experiments, we used two mouse models: CD-1 mice and mice deficient in interferon α/β receptor 1 (*Ifnar1^−/−^*). CD-1 and *Ifnar1^−/−^* mice were selected because our group has established these as susceptible models for USUV infection, with more severe disease in *Ifnar1^−/−^* mice [31,32]. Mice were vaccinated with D2/WN LAV and challenged with a European or African strain of USUV. CD-1 mice transiently treated with an anti-Ifnar1 antibody did not develop USUV disease but did have a neutralizing response to both WNV and USUV post-vaccination. We found that *Ifnar1^−/−^* mice vaccinated with D2/WN were protected against USUV-induced mortality and had lower USUV viremia than unvaccinated mice. Our results warrant further investigation into the mechanisms behind the cross-protection that WNV vaccination may provide against USUV.

## 2. Materials and Methods

### 2.1. Viruses and Cells

The D2/WN-V3 chimeric virus used in this study was a modified version of the D2/WN-V2 chimera that was described previously [33]. Briefly, D2/WN-V2 was constructed with the prM and E genes of the WNV NY99-35262 strain (GenBank AF196835) [34] in the backbone of the cDNA clone of the vaccine strain of D2 (PDK-53) (GenBank U87412.1) [33]. An additional Vero cell adaptation mutation at NS2A-22 (Met to Val) in the D2 backbone was engineered into the chimeric D2/WN-V2 clone for deriving a stable D2/WN-V3 for cell culture production; D2/WN-V3 raised similar immunogenicity and protected mice from lethal WNV challenge as the D2/WN-V2 LAV [26]. USUV strains used in these studies were HU10279-09 (USUV Spain 2009) [35] and UG09615 (USUV Uganda 2012) [36]. The USUV Spain 2009 isolate was passaged twice in Vero cells upon receipt, fully sequenced (GenBank MN813489), and characterized previously [32]. The USUV Uganda 2012 isolate was passaged four times in Vero cells and fully sequenced; the sequence is identical to a previous passage 3 sequence that was published (GenBank MN813491) [32]. Vero cells were grown at 37 °C and cultured in DMEM supplemented with 5% FBS and 1% penicillin-streptomycin.

### 2.2. Inoculation of Mice

#### 2.2.1. *Ifnar1^−/−^* Mice

**Figure 1:**
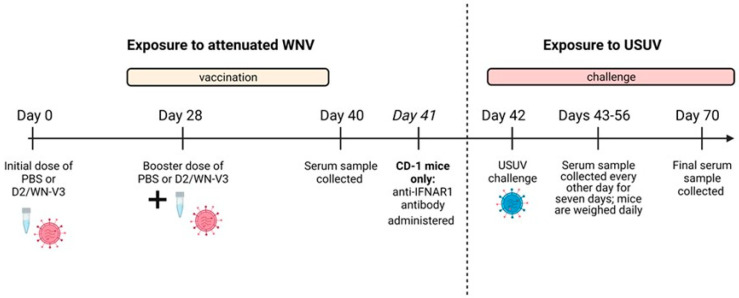
Study design. Studies using CD-1 (*n* = 40) and *Ifnar1^−/−^* (*n* = 32) mice were performed as indicated above. Study design was identical between two mouse models, with the exception of CD-1 mice receiving a dose of anti-IFNAR1 antibody to render them susceptible to USUV.

The interferon alpha and beta receptor 1 deficient mice *(Ifnar1^−/−^)* originally purchased from Jackson Laboratories (B6.129S2-*Ifnar1^tm1Agt^*/Mmjax) were bred in-house. A total of 32, 10 to18-weeks-old male and female mice were inoculated with 10^4^ PFU of D2/WN chimera (*n* = 16) or sterile PBS (*n* = 16) via intraperitoneal (i.p.) injection (Figure 1). Twenty-eight days post vaccination (DPV), mice received a second dose of D2/WN chimera at 10^4^ PFU, or PBS. A blood sample was collected via submandibular vein bleed 40 DPV. Forty-two days after the first vaccination, mice were challenged with 10^3^ PFU of USUV Spain 2009 isolate (*n* = 16) or USUV Uganda 2012 isolate (*n* = 16) via rear footpad injection, a method that was used previously for USUV [31,32]. Mice were bled via submandibular bleed on days 1, 3, 5, and 7 post-USUV challenge. Weights were taken daily, and mice were observed for clinical signs of illness (weight loss, lethargy, tremors). Mice were euthanized when exhibiting clinical signs of disease such as ≥15% weight loss, or at 28 days post-USUV challenge. Serum samples were titrated by Vero cell plaque assay.

#### 2.2.2. CD-1 Mice

A similar study was also performed in two independent experiments using 40, 8-weeks-old male and female CD-1 mice purchased from Charles River Laboratories (CD-1^®^ IGS); 20 mice were vaccinated with the WN/D2 chimera, and 20 mice received sterile PBS. Some modifications were made regarding dosage of the WN/D2 chimera, addition of a transient immunosuppressive antibody, and dosage of USUV. The amount of D2/WN administered on day 0 and day 28 was 10^5^ PFU. One day before USUV challenge (41 DPV) mice were transiently immunosuppressed with 1 mg of anti-mouse interferon α/β receptor purified function grade, GOLD monoclonal antibody (Clone MAR1-5A3, purchased from Leinco Technologies, Fenton, MO, USA, Inc; product # I-401) to render them susceptible to USUV infection (Figure 1). 42 days after the first vaccination, CD-1 mice were challenged with 10^5^ PFU of either the USUV Spain 2009 isolate (*n* = 19) or USUV Uganda 2012 isolate (*n* = 21). Mice were euthanized 28 days post-USUV challenge.

### 2.3. Plaque Reduction Neutralization Test (PRNT)

Mouse serum was heat-inactivated at 56 °C for 30 min. Serum was then serially diluted 2-fold in BA-1 diluent media (1X M199-Hank’s Salts w/o L-Glutamine, Sigma Chemical, St. Louis, MO, USA, product # M9163; 0.05M TRIS-HCl pH 7.5, Gibco, Waltham, MA, USA, product # 15567-027; 1% Bovine Serum Albumin, MilliporeSigma, Burlington, MA, USA, product # 81-066-4; 2 mM L-Glutamine, Invitrogen, Waltham, MA, USA, product # 25030-081; 0.35 g/L Sodium Bicarbonate, Gibco, Waltham, MA, USA, product # 25080-094; 100 units/mL Penicillin and 100 μg/mL Streptomycin, Gibco, Waltham, MA, USA,, product # 15140-122; 1μg/mL Amphotericin B, HyClone, HyClone, Logan, UT, USA, product # SV30078.01); an equal volume of BA-1 media containing approximately 100 PFU of virus (WNV or USUV) was added to each dilution. For USUV PRNTs, either HU10279-09 (USUV Spain 2009) or UG09615 (USUV Uganda 2012) was used depending on the in vivo challenge group of the sample. A negative control containing no serum was also included. Serum and virus mixtures were incubated at 37 °C for 1 h, then titrated by Vero cell plaque assay. The reciprocal serum dilution was recorded when the sample reduced plaque formation by at least 50% compared to that of the negative control.

### 2.4. Statistics

Changes in weight and serum titers were analyzed using a two-way ANOVA with Tukey’s multiple comparisons test. Survival curves between vaccinated and unvaccinated mice of the same USUV challenge group were analyzed using the Mantel–Cox test. Descriptive statistics were run to determine the geometric mean titers (GMTs) of vaccinated and unvaccinated groups for the PRNT_50_ results and compared via a nonparametric Mann–Whitney test. All analyses were done using GraphPad Prism 8.

### 2.5. Additional Software

The BioRender application was used to design Figure 1.

## 3. Results

### 3.1. Vaccinated CD-1 Mice Produce a Neutralizing Response against WNV and USUV Prior to USUV Challenge

**Figure 2:**
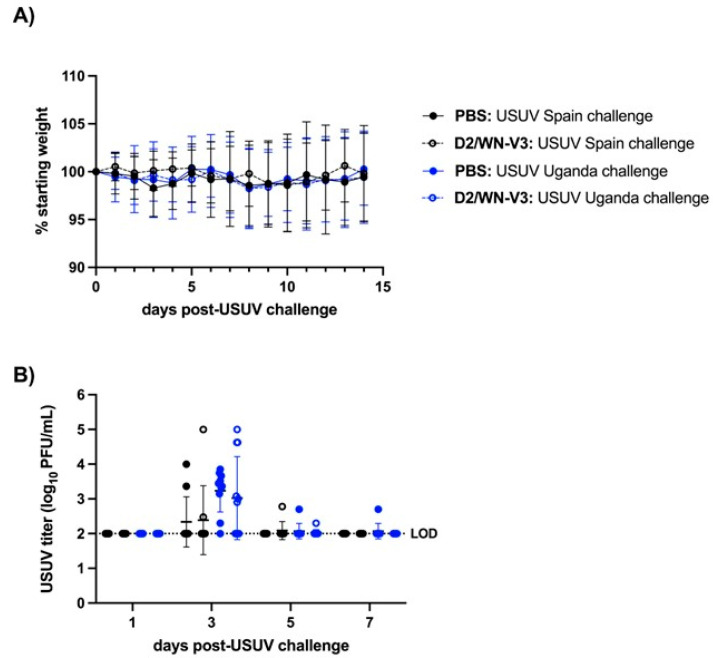
Morbidity and viremia profile of CD-1 mice post-USUV challenge. CD-1 mice were injected with D2/WN-V3 (*n* =20) or PBS (*n* = 20) and later challenged with a Spain (*n* = 19) or Uganda (*n* = 21) isolate of USUV. (**A**) Average percentage of initial weight post-challenge. (**B**) Viral titer of USUV in serum collected on day post-challenge (dpc) 1, 3, 5, and 7. Titers are reported as log_10_PFU per mL of serum. Lines represent mean; error bars represent standard deviation; and dashed line represents limit of detection (LOD). All negative titers were graphed at LOD and included in mean and standard deviation calculations.

To vaccinate immunocompetent mice against WNV, we used a D2/WN-V3 (D2/WN) LAV that was shown to confer protection against lethal WNV challenge [26]. CD-1 mice were given an initial dose of the vaccine or PBS and received a booster 28 days later (Figure 1). Mice were rendered susceptible to USUV with a dose of anti-IFNAR1 antibody prior to challenge, a strategy that was used for other wild-type mice subject to flavivirus infections including USUV [31,33,34]. Two recent USUV isolates from Spain and Uganda were used to challenge the vaccinated and PBS-treated mice on day 42 after the first vaccination. In this CD-1 mouse model of USUV infection, we did not observe any morbidity or weight loss; all mice survived and were euthanized at the experiment endpoint (28 days post-USUV challenge). No significant differences in weight change were observed between mock-vaccinated mice and vaccinated mice after USUV challenge (Figure 2A). Following a similar trend, no significant differences in viremia were observed between the vaccinated mice and unvaccinated mice (Figure 2B).

**Figure 3:**
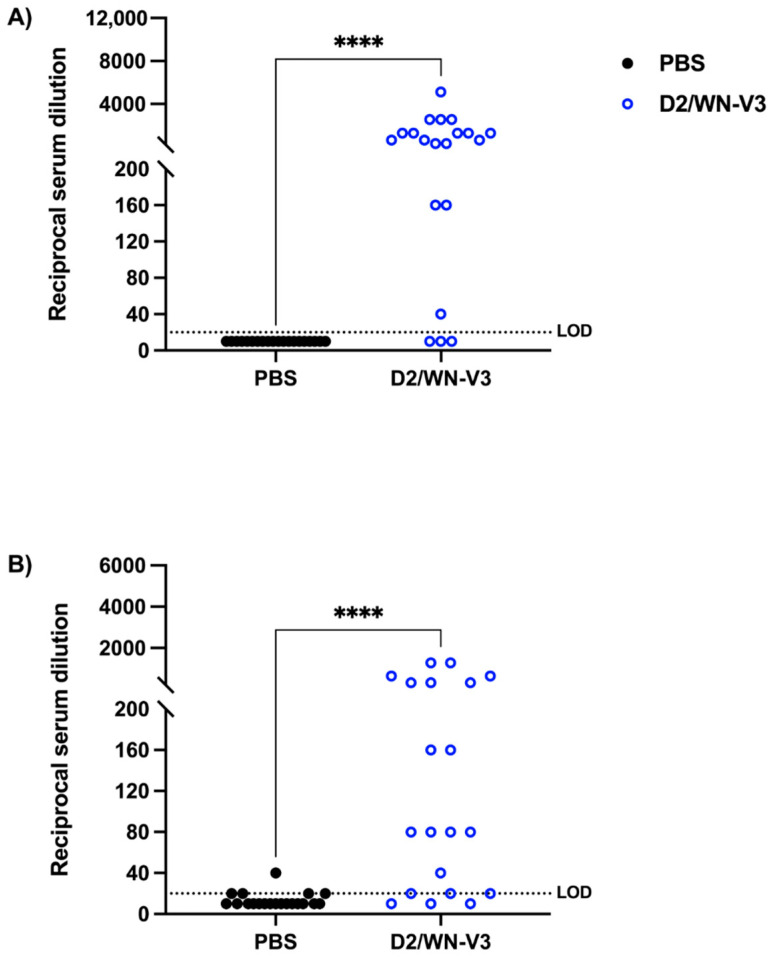
Neutralizing responses against WNV and USUV in CD-1 mice. Plaque reduction neutralization tests (PRNTs) against WNV and USUV were performed. Serum samples were collected postbooster (day 40 after initial vaccination) from CD-1 mice to determine geometric mean titers (GMTs) of each group. Each data point represents one serum sample. (**A**) GMTs of neutralizing responses against WNV. (**B**) GMTs of neutralizing responses against USUV. Data were collected from two independent experiments. Limit of detection (LOD) is 20. **** *p* < 0.0001. Negative samples (did not neutralize at least 50% of input virus at lowest dilution) were graphed at half LOD (10).

To determine whether exposure to an attenuated form of WNV would induce a neutralizing response against USUV, a serum sample was collected from each mouse prior to USUV challenge and plaque reduction neutralization tests (PRNTs) against WNV and USUV were performed. Mock-vaccinated mice did not produce a neutralizing response to WNV and produced a very low neutralizing response to USUV. D2/WN immunized mice had significantly higher PRNT_50_ titers against WNV than mock-vaccinated mice at a geometric mean titer (GMT) of 394 (*p* < 0.0001) (Table 1, Figure 3A). Interestingly, vaccinated mice also had significantly higher PRNT_50_ titers against USUV than mock-vaccinated mice at a GMT of 98.49 (*p* < 0.0001) (Table 1, Figure 3B). At day 28 post-USUV challenge, vaccinated mice had higher PRNT_50_ titers against WNV (744.9) compared to the pre-USUV challenge titers, though this difference was not significant.

### 3.2. WNV Vaccination Protects Ifnar1^−/−^ Mice from USUV Disease and Viremia

**Figure 4:**
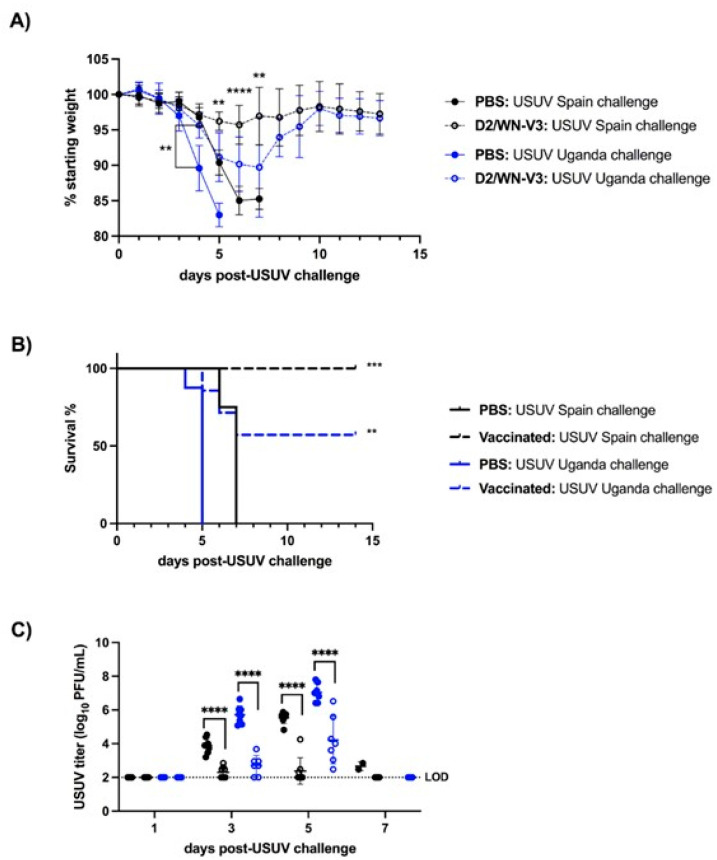
Morbidity, mortality, and viremia profile of *Ifnar1^−/−^* mice post-USUV challenge. *Ifnar1^−/−^* mice were treated with D2/WN-V3 (*n* = 16) or PBS (*n* = 16) and later challenged with a Spain (*n* = 16) or Uganda (*n* = 16) isolate of USUV. (**A**) Average percentage of initial weight post-challenge. *p*-values represent significant differences between D2/WN-V3 and PBS treated mice of same USUV challenge group. Day post-challenge (dpc) 4, ** *p* < 0.01 (Uganda challenge group); dpc 5, ** *p* < 0.01 (both challenge groups); dpc 6, **** *p* < 0.0001 (Spain challenge group); dpc 7, ** *p* < 0.01 (Spain challenge group) (**B**) Survival rate post-challenge. *** *p* < 0.001, ** *p* < 0.01 (D2/WN-V3 treated mice compared to that of PBS treated mice of same USUV challenge group); black lines represent USUV Spain challenge group and blue lines represent USUV Uganda challenge group; solid lines represent mice that received PBS and dashed lines represent mice that received D2/WN-V3. (**C**) Viral titer of USUV in serum collected on day post-challenge (dpc) 1, 3, 5, and 7. Titers are reported as log_10_PFU per mL of serum. Lines represent mean; error bars represent standard deviation; and dashed line represents limit of detection (LOD). All negative titers were graphed at LOD and included in mean and standard deviation calculations. **** *p* < 0.0001.

Next, we tested the vaccine in a mouse model that would develop USUV disease and high viremia levels. Our group has previously characterized the *Ifnar1 ^−/−^* as a suitable murine model for USUV infection with severe disease [32]. The same study design described for the CD-1 mice was used, with the omission of the anti-IFNAR1 antibody treatment. Mice that were mock-vaccinated with PBS lost weight quickly and succumbed to USUV infection by seven days post-challenge (dpc) (Figure 4A,B). Significant differences in survival between the two USUV challenge strains were observed in mock-vaccinated mice; mock-vaccinated mice challenged with the Uganda USUV isolate succumbed by 5 dpc while mock-vaccinated mice challenged with the Spain USUV isolate succumbed later (by 7 dpc, *p* < 0.0001) (Figure 4B). For both strains of USUV, vaccinated mice had significantly less weight loss at 5 dpc and higher survival rates than mock-vaccinated mice (Figure 4A,B). Vaccinated mice also had significantly lower USUV titers on 3 and 5 dpc compared to that of mock-vaccinated mice (*p* < 0.0001) (Figure 4C).

### 3.3. WNV Vaccination Induces a Neutralizing Response against WNV and USUV in Ifnar1^−/−^ Mice

**Figure 5:**
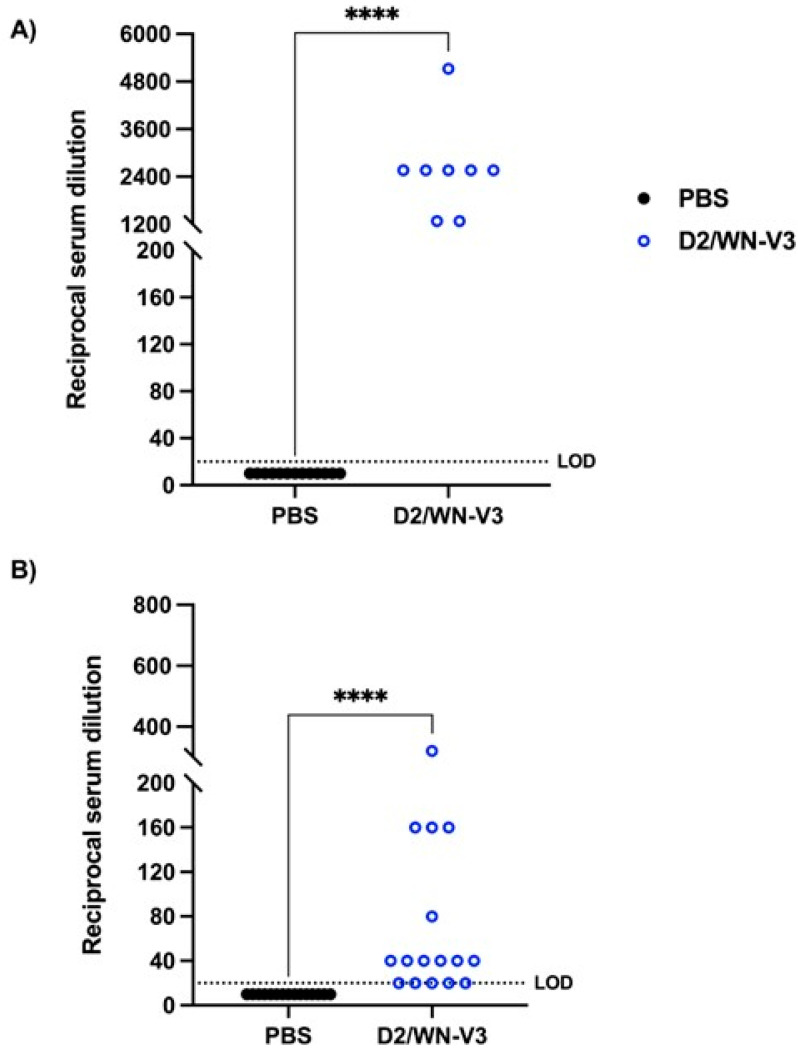
Neutralizing responses against WNV and USUV in *Ifnar1^−/−^* mice. Plaque reduction neutralization tests (PRNTs) against WNV and USUV were performed. Serum samples were collected post-booster (day 40 after initial vaccination) from *Ifnar1^−/−^* mice to determine geometric mean titers (GMTs) of each group. Each data point represents one serum sample. (**A**) GMTs of neutralizing responses against WNV. (**B**) GMTs of neutralizing responses against USUV. Due to inadequate sample volume, not all samples were tested against WNV. Limit of detection (LOD) is 20. **** *p* < 0.0001. Negative samples (did not neutralize at least 50% of input virus at lowest dilution) were graphed at half LOD (10).

To measure the neutralizing response against WNV and USUV before USUV challenge, a serum sample was collected from each mouse, and PRNTs against WNV and USUV were performed. Mock-vaccinated mice did not produce detectable neutralizing antibodies to either WNV or USUV. Sera from vaccinated mice neutralized both WNV and USUV, with a significantly higher geometric mean titer GMT of 2348 against WNV and 49.67 against USUV compared to that of mock-vaccinated mice (*p* < 0.0001) (Table 2, Figure 5).

## 4. Discussion

Through this study, we found that WNV vaccination induced a cross-reactive neutralizing response against USUV in mice (Table 1 and Table 2); these results were seen in two mouse strains, CD-1 mice and *Ifnar1^−/−^* mice, and two recent USUV strains from Spain and Uganda. Further, the WNV vaccine protected *Ifnar1^−/−^* mice against disease caused by USUV challenge and significantly reduced USUV viremia (Figure 4). The WNV vaccine did not reduce viremia in CD-1 mice, though USUV viremia in this mouse model was much lower than viremia in the *Ifnar1^−/−^* model (Figure 2). Our results indicate that a WNV vaccine induces a cross-neutralizing response against USUV in both mouse models, and that vaccination can protect against USUV mortality in *Ifnar1^−/−^* mice.

In this study, we did not observe USUV morbidity or mortality in adult CD-1 mice pre-treated with an IFNAR-blocking antibody (Figure 2A,B). In a previous study, we found that USUV infections with the Uganda 2012 strain resulted in USUV disease in some CD-1 mice pretreated with the IFNAR-blocking antibody, but these mice were only three-weeks old [31]; in this study, we used the same dose of IFNAR-blocking antibody (1mg/mouse), but the mice were 14-weeks-old at the time of USUV challenge. Additionally, in *Ifnar1^−/−^* mice we found that the WNV vaccine was less effective in mice challenged with the African strain (Uganda 2012 isolate) of USUV compared to the European (Spain 2009 isolate) strain of USUV. However, unvaccinated mice challenged with the Uganda 2012 USUV isolate succumbed earlier compared to mice challenged with the Spain 2009 USUV isolate (Figure 4B). The difference in pathogenesis between African and European USUV isolates was previously observed in the *Ifnar1^−/−^* model of USUV infection [32]. The difference in survival between vaccinated mice challenged with Uganda 2012 or Spain 2009 can thus likely be explained by the differential virulence of these strains, which is dictated by unknown viral genetic determinants. Notably, no disease was previously observed in newborn mice or AG129 mice with the WNV vaccine alone [26,30]. However, one limitation of this study is that tissues were not collected from mice to compare virus levels and pathology between vaccinated and unvaccinated mice. Previously, we saw high viral loads in the liver, spleen, heart, and brain in *Ifnar1^−/−^* mice infected with the Spain 2009 and Uganda 2012 USUV isolates, in addition to observable cell death and inflammation in the spleen [32].

Vaccination induced a more robust neutralizing response to WNV in *Ifnar1^−/−^* mice compared to CD-1 mice, indicated by the higher geometric mean antibody titers against WNV (Table 1 and Table 2). One possibility for this difference is that *Ifnar1^−/−^* mice are more susceptible to dengue viruses (DENVs) compared to that of immunocompetent mice [37,38]. In our study, the WNV vaccine was in a DENV2 replicative backbone, which likely limited the LAV replication efficiency in CD-1 mice, resulting in lower immunogenicity outcomes in this mouse model. For this reason, we used a higher dose of the WNV vaccine and USUV challenge in the CD-1 mice compared to the *Ifnar1^−/−^* mice. We also observed that vaccinated CD-1 mice mounted a higher neutralizing response to USUV compared to *Ifnar1^−/−^* mice (Table 1 and Table 2). One explanation for the difference in USUV neutralization between the two mouse models is that mice in a C57BL/6 background (the *Ifnar1^−/−^* model used here) are characterized by a high Th1 immune response, which corresponds to a dominantly cell-mediated immune response [39]. Thus, the cross-reactivity to USUV seen in vaccinated *Ifnar1^−/−^* mice may be driven by stronger cross-reactive T cells as opposed to cross-neutralizing antibodies. Although there was some USUV seroconversion in 5 mock vaccinated CD-1 mice, it was due to a low level of neutralization and did not influence the overall results, as there was still a highly significant difference in the neutralizing response against USUV between vaccinated and mock vaccinated mice (Table 1). We recognize that using the 50% neutralization threshold may cause variable results; however, this threshold is recommended by the WHO for flavivirus serology [40] and was used in the original paper characterizing the WNV vaccine [26].

The two-dose WNV vaccine-induced protection against USUV disease seen here is likely due at least partially to cross-reactive neutralizing antibodies. One of the primary targets for neutralizing antibodies against flaviviruses including WNV is the viral envelope (E) glycoprotein, which was included in the WNV vaccine used here. A variety of neutralizing antibodies against the E glycoprotein of WNV have been characterized in mice [41,42]; a humanized version of one of these antibodies, E16, was shown to protect mice from WNV infection [43]. A previous study showed that D2/WN induced a neutralizing response against WNV, with the second dose significantly boosting the immune response, and prevented mice from succumbing to lethal WNV challenge [26]. The two-dose WNV vaccination strategy was also implemented in AG129 mice, which are deficient in IFN-α/β and -γ responses, though there was less increase in the immune response in this mouse model from the second dose [30]. Our results confirmed that vaccinated mice developed a neutralizing response against WNV and a cross-reactive neutralizing response to USUV after two doses of the WNV vaccine (Table 1 and Table 2). Neutralizing antibodies against USUV are also likely important for USUV illness protection. One study showed a recombinant subviral particle vaccine containing USUV premembrane (prM) and E proteins protected *Ifnar1^−/−^* mice from lethal USUV challenge and induced a neutralizing antibody response [44].

It is also likely that some of the protection against USUV seen here is dependent on cellular immunity. Cell-mediated immune responses to WNV infection are also protective and can be directed against the E protein [45,46,47], which was included in the vaccine tested here. The important role of T cell immunity has been implicated in WNV, specifically in limiting infection of the central nervous system [45,46]. However, most dominant T cell epitopes of flaviviruses are located in the non-structural (NS) proteins [48], and the NS proteins expressed by the D2/WN LAV are from D2. Previous studies using D2/WN LAV have shown that WNV immunity elicited by the prM-E of the LAV provided 100% protection against lethal WNV challenge in mice, whereas D2 immunity generated by the NS proteins of the vaccine provided limited protection against lethal D2 challenge in an interferon-α/β/γ-receptor knockout AG129 mouse model [26,30]. Undoubtedly, both B and T cell immunity responses are important for protection against flaviviruses, but the neutralizing antibody response appears to play a dominant role in the disease protection in mouse models. Cross-reactive antibodies among some flaviviruses, especially different serotypes of DENV and possibly Zika virus, could result in cross-protection or disease enhancement outcome of the sequential heterotypic viral infection [49]. Currently, there is little evidence in immune enhancement of disease severity or virus infectivity between WNV and USUV infections. In contrast, cross-protection against WNV by prior USUV immunity was previously reported [50], and our study reports the first animal experiment data showing cross-protection against USUV illness by WNV vaccination.

WNV and USUV co-circulation also has the potential to impact both mosquito vectors and avian hosts. One mosquito surveillance study in Italy discovered numerous pools of *Culex pipiens* that tested positive for both WNV and USUV [51]. It was also shown that *Culex pipiens* preferentially transmit WNV when co-exposed to USUV and WNV via an infectious blood meal, though it is hypothesized that there is competition between viruses in the midgut of mosquitos [52]. Avian hosts also have the potential to be infected with both USUV and WNV. For instance, both WNV- and USUV-specific neutralizing antibodies were detected in migratory and resident birds in eastern Germany [53]. Recently, it was shown that magpies previously exposed to USUV are partially protected from WNV, indicated by higher survival rates post-WNV challenge [50]. The interactions between WNV and USUV in mosquito vectors and avian hosts will be critical to monitor as WNV and USUV continue to emerge.

Due to the continued emergence and spread of WNV globally [54], many efforts were made to further our understanding of WNV disease, particularly in developing murine models of infection. The similarities in emergence trends, antigenic properties, and geographic spread between WNV and USUV make studying these viruses in the context of one another an imperative process. Future passive and adoptive transfer studies could dissect protective efficacy by antibody- and cell-mediated immunity, respectively. Knowledge in cross-reactive immunity between WNV and USUV in their vertebrae host and mosquito vectors will be relevant information for predicting the potential public health impact of these emerging and re-emerging flaviviruses.

## Figures and Tables

**Table 1:** Neutralizing responses in CD-1 mice to WNV and USUV postvaccination.

	WNV PRNT50			USUV PRNT 50		
Treatment	GMT	% seroconverted	# mice	GMT	% seroconverted	# mice
PBS (mock-vaccinated)	10	0%	0/20	12.31	25%	5/20
D2/WN-V3 (vaccinated)	394 ****	85%	17/20	98.49 ****	85%	17/20

Plaque reduction neutralization tests (PRNTs) against WNV and USUV were performed. Serum samples were collected post-booster (day 40 after initial vaccination) from CD-1 mice to determine geometric mean titers (GMTs) of each group. Data were collected from two independent experiments. Limit of detection is 20. Negative samples (did not neutralize at least 50% of input virus) were assigned a value of 10 and included in GMT values of table. Statistical comparisons were done between mock-vaccinated and vaccinated groups for each virus; **** *p* < 0.0001 (vaccinated vs. mock-vaccinated).

**Table 2:** Neutralizing responses in *Ifnar1^−/−^* mice to WNV and USUV post-vaccination. Plaque reduction neutralization tests (PRNTs) against WNV and USUV were performed. Serum samples were collected post-booster (day 40 after initial vaccination) from *Ifnar1^−/−^* mice to determine geometric mean titers (GMTs) of each group. Limit of detection is 20. Negative samples (did not neutralize at least 50% of input virus) were assigned a value of 10 and included in GMT values of the table. Due to inadequate sample volume, not all samples were tested against WNV. Statistical comparisons were done between mock-vaccinated and vaccinated groups for each virus; **** *p* < 0.0001 (vaccinated vs. mock-vaccinated).

	WPN PRNT50			USUV PRNT50		
Treatment	GMT	% seroconverted	#mice	GMT	% seroconverted	#mice
PBS (mock-vaccinated)	10	0%	0/13	10	0%	0/15
D2/WN-V3 (vaccinated)	2348 ****	100%	8/8	49.67 ****	100%	16/16

## Data Availability

Data is available upon request.

## References

[1] Roesch F., Fajardo A., Moratorio G., Vignuzzi M. (2019). Usutu Virus: An Arbovirus on the Rise. Viruses.

[2] Smithburn K.H.T., Burke A. (1940). A neurotropic virus isolated from the blood of a native of Uganda. Am. J. Trop. Med. Hyg..

[3] Melnick J.L., Paul J.R., Riordan J.T., Barnett V.H., Goldblum N., Zabin E. (1951). Isolation from human sera in Egypt of a virus apparently identical to West Nile virus. Proc. Soc. Exp. Biol. Med..

[4] McIntosh B.M., Jupp P.G., Dos Santos I., Meenehan G.M. (1976). Epidemics of West Nile and Sindbis viruses in South Africa with Culex (Culex) univittatus Theobald as vector. S. Afr. J. Sci..

[5] Hurlbut H.S., Rizk F., Taylor R.M., Work T.H. (1956). A study of the ecology of West Nile virus in Egypt. Am. J. Trop. Med. Hyg..

[6] Chowdhury P., Khan S.A., Dutta P., Topno R., Mahanta J. (2014). Characterization of West Nile virus (WNV) isolates from Assam, India: Insights into the circulating WNV in northeastern India. Comp. Immunol. Microbiol. Infect. Dis..

[7] Myint K.S., Kosasih H., Artika I.M., Perkasa A., Puspita M., Ma’roef C.N., Antonjaya U., Ledermann J.P., Powers A.M., Alisjahbana B. (2014). West Nile virus documented in Indonesia from acute febrile illness specimens. Am. J. Trop. Med. Hyg..

[8] Li X.L., Fu S.H., Liu W.B., Wang H.Y., Lu Z., Tong S.X., Li Z.X., Nasci R.S., Kosoy O., Cui Y. (2013). West nile virus infection in Xinjiang, China. Vector Borne Zoonotic Dis..

[9] Russell R.C., Dwyer D.E. (2000). Arboviruses associated with human disease in Australia. Microbes Infect..

[10] Frost M.J., Zhang J., Edmonds J.H., Prow N.A., Gu X., Davis R., Hornitzky C., Arzey K.E., Finlaison D., Hick P. (2012). Characterization of virulent West Nile virus Kunjin strain, Australia, 2011. Emerg. Infect. Dis..

[11] Davis C.T., Ebel G.D., Lanciotti R.S., Brault A.C., Guzman H., Siirin M., Lambert A., Parsons R.E., Beasley D.W., Novak R.J. (2005). Phylogenetic analysis of North American West Nile virus isolates, 2001–2004: Evidence for the emergence of a dominant genotype. Virology.

[12] Ebel G.D., Carricaburu J., Young D., Bernard K.A., Kramer L.D. (2004). Genetic and phenotypic variation of West Nile virus in New York, 2000–2003. Am. J. Trop. Med. Hyg..

[13] Morales M.A., Barrandeguy M., Fabbri C., Garcia J.B., Vissani A., Trono K., Gutierrez G., Pigretti S., Menchaca H., Garrido N. (2006). West Nile virus isolation from equines in Argentina, 2006. Emerg. Infect. Dis..

[14] Bernkopf H., Levine S., Nerson R. (1953). Isolation of West Nile virus in Israel. J. Infect. Dis..

[15] Joubert L., Oudar J., Hannoun C., Beytout D., Corniou B., Guillon J.C., Panthier R. (1970). Epidemiology of the West Nile virus: Study of a focus in Camargue. IV. Meningo-encephalomyelitis of the horse. Ann. Inst. Pasteur..

[16] Hannoun C., Panthier R., Mouchet J., Eouzan J.P. (1964). Isolation in France of the West Nile Virus from Patients and from the Vector Culex Modestus Ficalbi. Comptes Rendus Hebd. Seances Acad. Sci..

[17] Williams M.C., Simpson D.I., Haddow A.J., Knight E.M. (1964). The Isolation of West Nile Virus from Man and of Usutu Virus from the Bird-Biting Mosquito Mansonia Aurites (Theobald) in the Entebbe Area of Uganda. Ann. Trop. Med. Parasitol..

[18] Engel D., Jost H., Wink M., Borstler J., Bosch S., Garigliany M.M., Jost A., Czajka C., Luhken R., Ziegler U. (2016). Reconstruction of the Evolutionary History and Dispersal of Usutu Virus, a Neglected Emerging Arbovirus in Europe and Africa. mBio.

[19] Luhken R., Jost H., Cadar D., Thomas S.M., Bosch S., Tannich E., Becker N., Ziegler U., Lachmann L., Schmidt-Chanasit J. (2017). Distribution of Usutu Virus in Germany and Its Effect on Breeding Bird Populations. Emerg. Infect. Dis..

[20] Weissenbock H., Kolodziejek J., Url A., Lussy H., Rebel-Bauder B., Nowotny N. (2002). Emergence of Usutu virus, an African mosquito-borne flavivirus of the Japanese encephalitis virus group, central Europe. Emerg. Infect. Dis..

[21] Pierro A., Gaibani P., Spadafora C., Ruggeri D., Randi V., Parenti S., Finarelli A.C., Rossini G., Landini M.P., Sambri V. (2013). Detection of specific antibodies against West Nile and Usutu viruses in healthy blood donors in northern Italy, 2010–2011. Clin. Microbiol. Infect..

[22] Faggioni G., De Santis R., Pomponi A., Grottola A., Serpini G.F., Meacci M., Gennari W., Tagliazucchi S., Pecorari M., Monaco F. (2018). Prevalence of Usutu and West Nile virus antibodies in human sera, Modena, Italy, 2012. J. Med. Virol..

[23] Sinigaglia A., Pacenti M., Martello T., Pagni S., Franchin E., Barzon L. (2019). West Nile virus infection in individuals with pre-existing Usutu virus immunity, northern Italy, 2018. Eurosurveillance.

[24] Aberle S.W., Kolodziejek J., Jungbauer C., Stiasny K., Aberle J.H., Zoufaly A., Hourfar M.K., Weidner L., Nowotny N. (2018). Increase in human West Nile and Usutu virus infections, Austria, 2018. Eurosurveillance.

[25] Blazquez A.B., Escribano-Romero E., Martin-Acebes M.A., Petrovic T., Saiz J.C. (2015). Limited susceptibility of mice to Usutu virus (USUV) infection and induction of flavivirus cross-protective immunity. Virology.

[26] Huang C.Y., Silengo S.J., Whiteman M.C., Kinney R.M. (2005). Chimeric dengue 2 PDK-53/West Nile NY99 viruses retain the phenotypic attenuation markers of the candidate PDK-53 vaccine virus and protect mice against lethal challenge with West Nile virus. J. Virol..

[27] Butrapet S., Huang C.Y., Pierro D.J., Bhamarapravati N., Gubler D.J., Kinney R.M. (2000). Attenuation markers of a candidate dengue type 2 vaccine virus, strain 16681 (PDK-53), are defined by mutations in the 5′ noncoding region and nonstructural proteins 1 and 3. J. Virol..

[28] Yoksan S., Bhamarapravati N., Halstead S.B., St. George T.D., Kay B.H., Block J. (1986). Dengue virus vaccine development: Study on biological markers of uncloned dengue 1–4 viruses serially passaged in primary kidney cells, Arbovirus Research in Australia. Proceedings of the 4th Symposium CSIRO/QIMR.

[29] Huang C.Y., Butrapet S., Tsuchiya K.R., Bhamarapravati N., Gubler D.J., Kinney R.M. (2003). Dengue 2 PDK-53 virus as a chimeric carrier for tetravalent dengue vaccine development. J. Virol..

[30] Calvert A.E., Huang C.Y., Kinney R.M., Roehrig J.T. (2006). Non-structural proteins of dengue 2 virus offer limited protection to interferon-deficient mice after dengue 2 virus challenge. J. Gen. Virol..

[31] Bates T.A., Chuong C., Hawks S.A., Rai P., Duggal N.K., Weger-Lucarelli J. (2021). Development and characterization of infectious clones of two strains of Usutu virus. Virology.

[32] Kuchinsky S.C., Hawks S.A., Mossel E.C., Coutermarsh-Ott S., Duggal N.K. (2020). Differential pathogenesis of Usutu virus isolates in mice. PLoS Negl. Trop. Dis..

[33] Kinney R.M., Butrapet S., Chang G.J., Tsuchiya K.R., Roehrig J.T., Bhamarapravati N., Gubler D.J. (1997). Construction of infectious cDNA clones for dengue 2 virus: Strain 16681 and its attenuated vaccine derivative, strain PDK-53. Virology.

[34] Lanciotti R.S., Roehrig J.T., Deubel V., Smith J., Parker M., Steele K., Crise B., Volpe K.E., Crabtree M.B., Scherret J.H. (1999). Origin of the West Nile virus responsible for an outbreak of encephalitis in the northeastern United States. Science.

[35] Vazquez A., Ruiz S., Herrero L., Moreno J., Molero F., Magallanes A., Sánchez-Seco P.M., Figuerola J., Tenorio A. (2011). West Nile and Usutu viruses in mosquitoes in Spain, 2008–2009. Am. J. Trop. Med. Hyg..

[36] Mossel E.C., Crabtree M.B., Mutebi J.P., Lutwama J.J., Borland E.M., Powers A.M., Miller B.R. (2017). Arboviruses Isolated From Mosquitoes Collected in Uganda, 2008–2012. J. Med. Entomol..

[37] Shresta S., Kyle J.L., Snider H.M., Basavapatna M., Beatty P.R., Harris E. (2004). Interferon-dependent immunity is essential for resistance to primary dengue virus infection in mice, whereas T- and B-cell-dependent immunity are less critical. J. Virol..

[38] Johnson A.J., Roehrig J.T. (1999). New mouse model for dengue virus vaccine testing. J. Virol..

[39] Mills C.D., Kincaid K., Alt J.M., Heilman M.J., Hill A.M. (2000). M-1/M-2 macrophages and the Th1/Th2 paradigm. J. Immunol..

[40] World Health Organization (2007). Guidelines for Plaque Reduction Neutralization Testing of Human Antibodies to Dengue Viruses.

[41] Oliphant T., Nybakken G.E., Austin S.K., Xu Q., Bramson J., Loeb M., Throsby M., Fremont D.H., Pierson T.C., Diamond M.S. (2007). Induction of epitope-specific neutralizing antibodies against West Nile virus. J. Virol..

[42] Nybakken G.E., Oliphant T., Johnson S., Burke S., Diamond M.S., Fremont D.H. (2005). Structural basis of West Nile virus neutralization by a therapeutic antibody. Nature.

[43] Oliphant T., Engle M., Nybakken G.E., Doane C., Johnson S., Huang L., Gorlatov S., Mehlhop E., Marri A., Chung K.M. (2005). Development of a humanized monoclonal antibody with therapeutic potential against West Nile virus. Nat. Med..

[44] Martin-Acebes M.A., Blazquez A.B., Canas-Arranz R., Vazquez-Calvo A., Merino-Ramos T., Escribano-Romero E., Sobrino F., Saiz J.C. (2016). A recombinant DNA vaccine protects mice deficient in the alpha/beta interferon receptor against lethal challenge with Usutu virus. Vaccine.

[45] Shrestha B., Diamond M.S. (2004). Role of CD8+ T cells in control of West Nile virus infection. J. Virol..

[46] Shrestha B., Samuel M.A., Diamond M.S. (2006). CD8+ T cells require perforin to clear West Nile virus from infected neurons. J. Virol..

[47] Sitati E.M., Diamond M.S. (2006). CD4+ T-cell responses are required for clearance of West Nile virus from the central nervous system. J. Virol..

[48] Hassert M., Brien J.D., Pinto A.K. (2019). Mouse Models of Heterologous Flavivirus Immunity: A Role for Cross-Reactive T Cells. Front. Immunol..

[49] Katzelnick L.C., Bos S., Harris E. (2020). Protective and enhancing interactions among dengue viruses 1-4 and Zika virus. Curr. Opin. Virol..

[50] Escribano-Romero E., Jimenez de Oya N., Camacho M.C., Blazquez A.B., Martin-Acebes M.A., Risalde M.A., Muriel L., Saiz J.C., Hofle U. (2021). Previous Usutu Virus Exposure Partially Protects Magpies (Pica pica) against West Nile Virus Disease But Does Not Prevent Horizontal Transmission. Viruses.

[51] Calzolari M., Bonilauri P., Bellini R., Albieri A., Defilippo F., Maioli G., Galletti G., Gelati A., Barbieri I., Tamba M. (2010). Evidence of simultaneous circulation of West Nile and Usutu viruses in mosquitoes sampled in Emilia-Romagna region (Italy) in 2009. PLoS ONE.

[52] Wang H., Abbo S.R., Visser T.M., Westenberg M., Geertsema C., Fros J.J., Koenraadt C.J.M., Pijlman G.P. (2020). Competition between Usutu virus and West Nile virus during simultaneous and sequential infection of Culex pipiens mosquitoes. Emerg. Microbes. Infect..

[53] Michel F., Sieg M., Fischer D., Keller M., Eiden M., Reuschel M., Schmidt V., Schwehn R., Rinder M., Urbaniak S. (2019). Evidence for West Nile Virus and Usutu Virus Infections in Wild and Resident Birds in Germany, 2017 and 2018. Viruses.

[54] Pierson T.C., Diamond M.S. (2020). The continued threat of emerging flaviviruses. Nat. Microbiol..

